# Autistic Perspectives on the Future of Clinical Autism Research

**DOI:** 10.1089/aut.2022.0017

**Published:** 2022-06-09

**Authors:** Heta Pukki, Jorn Bettin, Avery Grey Outlaw, Joshua Hennessy, Kabie Brook, Martijn Dekker, Mary Doherty, Sebastian C.K. Shaw, Jo Bervoets, Silke Rudolph, Thibault Corneloup, Kylieanne Derwent, Onemoo Lee, Yadira Garcia Rojas, Wenn Lawson, Monica Vidal Gutierrez, Kosjenka Petek, Myria Tsiakkirou, Annikka Suoninen, Jo Minchin, Rainer Döhle, Silke Lipinski, Heini Natri, Emma Reardon, Giovanna Villarreal Estrada, Ovidiu Platon, Nick Chown, Ayaya Satsuki, Damian Milton, Nick Walker, Ondrej Roldan, Bárbara Herrán, Citlali Limón Cañedo, Sue McCowan, Mona Johnson, Eleanor Jane Turner, Jessy Lammers, wn-ho Yoon

**Affiliations:** ^1^ European Council of Autistic People z.s., Prague, Czech Republic.; ^2^ Autistic Collaboration Trust, Auckland, New Zealand.; ^3^ Autistic Self-Advocacy Network, Washington, District of Columbia, USA.; ^4^ Autism Rights Group Highland, Inverness, Scotland, UK.; ^5^ European Council of Autistic People z.s, Prague, Czech Republic.; ^6^ Autistic Doctors International, Dublin, Ireland.; ^7^ Autistic Doctors International, United Kingdom.; ^8^ Lees- en Adviesgroep Volwassenen Autisme vzw, Antwerpen, Belgium.; ^9^ European Council of Autistic People z.s, Prague, Czech Republic.; ^10^ CLE Autistes, Paris, France.; ^11^ The Autistic Realm, Nowra Hill, Australia.; ^12^ estas, Seoul, Korea.; ^13^ Autistas de México, A.C., Ciudad de Mexico, México.; ^14^ Independent, Warrnambool, Australia.; ^15^ Mi Cerebro Atípico/Asociación Autistas de Colombia, Bogota, Colombia.; ^16^ Incijativa za autizam i ostale neurodivergentnosti, Zagreb, Croatia.; ^17^ European Council of Autistic People z.s, Prague, Czech Republic.; ^18^ Suomen Autismikirjon Yhdistys ry., Helsinki, Finland.; ^19^ The National Autistic Taskforce, Lincoln, United Kingdom.; ^20^ Aspies e.V., Berlin, Germany.; ^21^ Autismus-Forschungs-Kooperation, Berlin, Germany.; ^22^ The Translational Genomics Research Institute, Phoenix, Arizona, USA.; ^23^ Autism Wellebing CIC, Carmarthen, Wales, UK.; ^24^ Asociația suntAutist—Autismul explicat de autiști, Timisoara, Romania.; ^25^ Independent Autism Research Group, Cradley Heath, United Kingdom.; ^26^ Otoemojite Neurodiversity Self-Help Group, Tokyo, Japan.; ^27^ The Participatory Autism Research Collective, Canterbury, United Kingdom.; ^28^ California Institute of Integral Studies, San Francisco, California, USA.; ^29^ Adventor, Prague, Czech Republic.; ^30^ Mi Cerebro Atípico, Lima, Perú.; ^31^ Independent, Jalisco, México.

**Keywords:** autistic people's priorities, autism research, collaborative participation, neurodiversity paradigm, participatory research

The *Lancet* Commission on the *Future of Care and Clinical Research in Autism* recently published their recommendations for what should be done in the next 5 years to address the current needs of autistic individuals and families.^1^ Although the Commission includes many prominent clinicians and researchers from around the world, as well as some autistic advocates and parents of autistic people, there have been widespread expressions of dissatisfaction among autistic people and communities regarding these recommendations.

We, the *Global Autistic Task Force on Autism Research* are a group of autistic professionals and representatives of organizations run by and for autistic people. We are autistic clinicians, therapists, educators and researchers, parents, and family members of autistic people of all ages and with all types of support needs, as well as individuals with high support needs. Among us are also autistic people of color, autistic people from the Global South and Asia, autistic women, and autistic people belonging to gender minorities.

Despite aiming at bringing together different stakeholders, representation within the Lancet Commission was limited in these respects. We hope to bring more voices to the discourse. We previously wrote an open letter to the Commission to draw attention to our main concerns.^2^ In this editorial, we offer a more detailed discussion of the Commission's report, as well as our own recommendations for future directions in autism research and care.

The Commission gives detailed recommendations on the types of studies and the research themes they consider most important for funding, highlighting randomized controlled trials for short-term interventions, including medication and behavioral trials, as a priority. The Commission recommends more research on the usability of diagnostic methods and practices in different countries, and it considers it necessary to bring about system change, by which they mean making local and national systems more effective at delivering diagnostic services and interventions.

In addition, the Commission proposes a classification of “profound autism” be adopted as an administrative term to apply to autistic children and adults with high support needs, for example, those with a co-occurring intellectual disability or limited ability to communicate by using spoken language.

The Commission endorses research on genetics, biomarkers, and the development of medications to treat autism as well as co-occurring conditions, but it stresses the importance of investing resources in research focusing on practical clinical approaches, strategies, and treatments that can be implemented immediately to yield faster results. To implement the various clinical approaches effectively, the Commission proposes a “stepped care and personalized health model for interventions in autism.”

The proposed model starts with identifying the relevant diagnoses or conditions that require services, prioritizing needs, and defining the goals of treatments. The model also involves considering individual and family factors that may affect treatment success, as well as the accessibility and cost of the various interventions. These interventions are described as focusing on “building skills that are absent or diminished,” such as neurotypical social skills, as well as “reducing behaviors or feelings that have negative effects,” such as temper tantrums, aggression, depressive feelings, irritability, or hyperactivity.

Also, the Commission highlights as one of their key messages that “valuing autism and neurodiversity benefits society as a whole,” and it argues that neurodiversity, along with other factors, is “important for an understanding of any autistic individual and of the differences between individuals who have this diagnosis.”

We believe that the report falls short of truly including autistic perspectives. We focus on several key concerns: (1) We feel the report inadequately incorporates the advocacy and scholarship of autistic people and misunderstands the neurodiversity paradigm; (2) we consider the functional classification of “profound autism” to be misleading and counterproductive; and (3) we point out that the Commission's recommendations are in certain respects incomplete and, therefore, risk misrepresenting the necessary priorities for the next 5 years.

We close with a call to action, based on recent research on participatory approaches, in which we propose to set up true collaborative efforts in the spirit of the Commission's proposals, including our autistic perspective from the outset rather than as a mere output quality check.

## Limited Consideration of the Advocacy and Scholarship of Autistic People

We find it encouraging that the Lancet Commission mentions the importance of collaborative participation when discussing the future of autism research, and we look forward to being increasingly included as collaborators.

We also find it positive that the Lancet Commission recognizes the need for systemic change and participatory research with all stakeholders, as well as the need for quality standards in autism research with randomized trials. Similarly, it is positive that the report discusses the urgent needs of autistic adults, although briefly, and that there were some autistic members in the Commission.

Nevertheless, it seems that as autistic adults and producers of knowledge on autism, the vast majority of autistic people in general, as well as autistic researchers, have remained invisible. For example, key studies and reports mapping autistic people's priorities regarding autism research have not been cited.^3–8^ These provide some broader context: Most autistic people's primary wishes for the next 5 years would not concern clinical interventions but matters of law, ethics, policy, and how these translate into support practices and realization of human rights.^3^

More than three decades of individual and collective advocacy, scholarship, and development of theory and praxis by autistic people were covered by the Commission under the heading of parent and family advocacy, in three words: “increasingly, self-advocacy.” Autistic people who offer their expertise and experience-based knowledge appear to be generalized as “more able” despite our widely varying support needs, and despite many of our organizations focusing partly or primarily on the needs of those who are less able to advocate for themselves.^9–11^ Similarly, we appear to be grouped under the title of the neurodiversity movement, despite our different approaches and varying levels of emphasis on the concept, and despite the history of the autistic rights movement preceding it.^12,13^

Regarding the neurodiversity paradigm, we wish to point out that considering something as natural variation does not equal claiming that it “does not need intervention.” It means preferring interventions that target systems and environments, supporting individuals to thrive as they are, instead of trying to bring them closer to the “perceived norm.”^14–19^ We agree with the Commission's observation that “not all autistic people and stakeholders identify with the neurodiversity movement.” We would welcome research on the distribution of people identifying with the neurodiversity movement or paradigm versus identifying with clinical and other portrayals of autism.

We also wish to point out that researchers embracing the neurodiversity paradigm do not comprise a new phenomenon.^20^ It has been encouraging to see the increasing number of established autism researchers re-considering the traditional framing of autism and recommending this approach to colleagues.^21,22^

On a fundamental level, we need to be seen from the dual perspectives of minority and disability. We find it positive that the Lancet Commission suggests using the International Classification of Functioning, Disability and Health in research. However, the authors consistently refer to autism as a “disorder” rather than a “disability,” while equally consistently using the expressions “intellectual disability” and “learning disability.” This terminology appears to signal that autism itself belongs to the category of illness or disease, rather than neurodivergence or disability, both of which would allow for the inclusion of positive characteristics as part of the core definition of autism.^23^

## The Term “Profound Autism”

The Commission proposes the label of “profound autism” to be adopted as a term to apply to autistic children and adults who have or are likely to have particular support needs, specifically, those “requiring 24 hours access to an adult who can care for them if concerns arise, being unable to be left completely alone in a residence, and not being able to take care of basic daily adaptive needs.” The commission notes that in most cases, these needs will be associated with a substantial intellectual disability, very limited language, or both, effectively creating a label to classify the most vulnerable autistic individuals. The Commission further states that the term “profound autism” was chosen as the term “low-functioning” is disliked by many.

We do not agree with the proposal to adopt “profound autism” as an administrative term. For more than 30 years, autistic people have resisted functioning labels as misleading and offensive.^24,25^ “Profound autism” would be a step back, even as “low-functioning” is falling out of use.

The term would not be sufficient to steer service provision or research efforts, just as functioning levels never were. It provides no useful information to others who may need to interact with the autistic person. High support needs are associated with co-occurring characteristics and health issues in many combinations, and the level of support needs often fluctuates. It is clearer to use brief descriptions such as “autistic person with intellectual disability,” “autistic person with minimal language,” or “autistic person with extreme anxiety and co-occurring physical condition.” Expressions such as “autistic person with high support needs” or “autistic person requiring 24-hour care” are also useful.

The term would also give the false impression of intellectual disability and impaired language development being core characteristics of autism. An autistic person with these characteristics would somehow be “more autistic,” or closer to the deep end of an imaginary linear spectrum, than an autistic person without them. “Profoundly autistic” would misleadingly refer to people who actually have *profound impairments* that are not autism specific, while not necessarily having any particularly extreme autistic characteristics.

## Participatory Research

Participatory research gets two brief mentions in the Commission report. The Commission states that research should in all contexts embrace a participatory approach that includes autistic people, alongside other stakeholder groups. This stated importance is not reflected in the rest of the report, as no definitions of good examples of participatory research are offered, and only one citation is provided. The only examples of participatory roles that the Commission suggests for autistic people in the context of clinical research are “consulting on the details of clinical trials and outcome measures.”

The participatory approach is a crucial element in all future autism research. A body of literature exists on its principles, practices, and significance.^26–33^ Anything that will truly help needs to be co-designed, developed, and evaluated with the involvement of autistic people. It has positive implications for the wider research agenda, in particular when established non-autistic autism researchers collaborate meaningfully with autistic scholars. We need approaches that value and center autistic voices, experiences, and expertise.

As participants, autistic people can correct misperceptions regarding concepts developed by autistic communities, researchers, and scholars, including neurodiversity and the neurodiversity paradigm,^34–36^ the double empathy problem,^37^ autistic inertia,^38^ monotropism,^39^ hyperfocus,^40^ and autistic space.^41^ We can offer insights on the therapeutic and empowerment value of self-help activities and the positive aspects of engaging in intense interests, as well as introducing emerging ideas such as sensory trauma,^42^ the co-creation of extended autistic families, and community-based mentoring.^43^ These concepts have implications for clinical research, including early intervention,^44^ and can lead research to new, more effective directions.

## Research Topics and Approaches

The Lancet Commission highlights randomized controlled trials for short-term interventions, including medication and behavioral interventions, as a priority in clinical autism research. The Commission emphasizes the need for trials to test the relative efficacy of different types, intensities, and combinations of interventions, as well as assessing the generalization of particular interventions and moderators of response and outcome.

Although the Commission states that the inclusion of stakeholders in the development of trial designs and outcome measures is vital, we do not believe that the Commission's recommendations regarding clinical trials adequately consider the needs and well-being of autistic people. As such, we do not believe that the Lancet Commission's recommendations can lead to improvements in the lives of autistic people, or the lives of our autistic children, within the next 5 years.

Instead, we urge focusing more resources on causes of mortality, improving our access to health care^45–49^ and mental health support,^50–53^ and large-scale monitoring of the effects of better access. This should include the impact on the detection and treatment of co-occurring conditions. We find it particularly surprising that the mortality studies published over the past few years received very little attention in recommendations for clinical research.^54–58^

We need clinical research to tackle the problem of harmful pseudoscientific treatments, mapping their use and effects on mental and physical health, as well as improving awareness among clinicians and carers.^59^

We need more research on assisted and augmented communication and supported decision making in the context of clinical work and care. To autistic people with limited communication, they are crucial to accessing health care and the appropriate delivery of medical and care services. Advocacy organizations often appear to be needed to ensure the right to use them, when this should be part of the work of clinical and care staff.^60,61^

We need more research on how stressful environments, being misperceived by others, lack of appropriate social and disability services, lack of reasonable accommodations, stigma, discrimination, and bullying affect the health of autistic people, contributing to anxiety and depression, and how such effects can be mitigated.^62–66^ A counseling methodology for autistic and other neurodivergent people needs to be developed and tested.

We also urgently need research on large-scale, affordable, and accessible screening and diagnosis for all age groups, in all parts of the world, especially in low-income and middle-income countries (LMICs).^67,68^ Diagnostic services must be seen as a basic right for all autistic people, not something that clinicians grant at their discretion or only when the diagnosis can lead to the provision of interventions. Diagnosis helps both children and adults to develop identity, self-knowledge, and personal strategies, and to start engaging in peer support, self-advocacy, and collective advocacy. These can be crucial to well-being, quality of life, and realization of basic rights, especially when very little else is available.^69^

The Commission has recognized the importance of research in adolescents and adults. However, we find that the recommendations for clinical research fall short, offering no concrete suggestions for topics or types of research. Repeating that something is urgently needed does not provide sufficient guidance.

The social^70^ and human rights^71^ models of disability should be understood and applied in the context of clinical work, leading to research and practice models that target systems, not just us as individuals. The existence of interventions with the goal of “optimizing person-environment fit” has been recognized by the Commission, which is a promising first step, but again this is not linked to concrete recommendations. Studies focusing on the Double Empathy Problem^37^ and unconscious negative perceptions of autistic people^16,63^ provide both theoretical frameworks and examples of practical interventions.^16,17,44^

Some aspects of the Treatment and Education of Autistic and related Communications Handicapped Children (TEACCH) programme, covered by the Commission in two sentences, might also offer starting points as a decades-old approach focused on adjusting environments.^72^ Educational and employment interventions need to be developed that promote positive uses of autistic people's intense interests and capacity for passionate focus, seeing the potential at the system level. They should not be dismissed as signs of “restricted” thinking or limited to being used in teaching social interaction as happens in the Program for the Education and Enrichment of Relationship Skills (PEERS), which the commission consistently recommends.^1^

Studying system change may not be amenable to controlled trials or commodification of intervention services. This does not diminish its significance. We need clinical research to work in collaboration with other fields of autism research, becoming part of a fundamental cultural shift in approaching autism, instead of falling outside it and operating in isolation.

## Addressing Harmful Research and Treatments

The Lancet Commission discusses the problem of pseudo-treatments being promoted in popular literature and on the Internet, using the term “non-evidence-based treatments,” and stressing the responsibility of clinicians to be informed on which treatments are evidence-based, to be able to guide and advise caregivers.

However, we feel that some important aspects of this topic did not receive enough attention. Autistic children, adolescents, and adults with limited ability to advocate for themselves need to be actively protected from malpractice.^59^

Early autism research often objectified autistic people, and in many cases caused immense harm. Unfortunately, this can still happen. Historically, autistic children and dependent adults who are unable to give informed consent have been enrolled in experiments that may not have been allowed on non-autistic people, based on weak, far-fetched hypotheses. This has fed the creation of new pseudo-treatments. Research that focuses on pseudo-cures decenters the voices of autistic people regarding our actual needs and priorities.

We wish to draw attention to the fact that many pseudo-treatments have been initially trialed at universities and other research institutions, or promoted by them, including “packing,”^73,74^ holding therapy,^75^ secretin,^76^ hyperbaric oxygen,^77^ fecal transplants,^78,79^ oxytocin,^80^ and injections of stem cells,^81^ to name only a few. Clinical trials can be driven by the promise of commercializing a new “solution,” and by negative bias and disdain toward autistic people. It is necessary to recognize that some structures and practices of the academic world allow or even support the development of pseudo-treatments, which seek a thin veil of apparent academic credibility to attract followers and funding.

Autistic people's organizations have attempted to draw attention to the fact that defining the behavioral characteristics listed in diagnostic criteria as “core,” and trying to develop biological treatments with behavior as the primary target and means of measuring success, is both unwanted by many autistic people and likely destined to fail; similar behavior does not equal similar biology.^82^ The inherent fallacy in this approach is likely to contribute to continuous generation of pseudo-treatments. We urge researchers to focus the development of medications on co-occurring health problems that autistic people identify as distressing, and to target clearly identifiable biological factors instead of behaviors, with the aim of improving autistic people's quality of life and appreciating neurodiversity in this context as well as others. We predict that for many of us, better health would automatically lead to some positive changes in cognition and behavior as well as quality of life and well-being.

## Behavioral Concepts and Interventions

We are particularly concerned about the dominant role of behavioral interventions, concepts, and interpretations of autism that is evident throughout the Commission's text. For example, the word “behavior” appears in the Commission's publication 161 times; in contrast, the word “cognitive” only appears 32 times, “quality of life” 12 times, “sensory” 9 times, and “wellbeing” only 4 times. The Commission endorses the use of behavioral interventions to target autistic behavioral and social communication differences and underlines conducting randomized controlled trials for short-term interventions, including behavioral trials, as a priority.

Autistic-led organizations have engaged in widespread criticism, activism, and campaigning focusing on behavioral approaches, including recent appeals to the UN Committee on the Rights of People with Disabilities.^83^ We see this especially in countries where the methods have been used extensively.

Changing behavior, as such, should not be the main goal of clinical research or treatment for autistic people of any age. Appearing autistic or acting in typically autistic ways should not be considered an illness. Clinicians need to be aware of the potential mental health risks of “camouflaging” and avoid encouraging or manipulating autistic people to engage in it, even through naturalistic or play-based methods.^84,85^ Keeping in mind health and well-being as the goals of clinical work, and the fundamental principle of beneficence, research should explore the long-term effects of behavioral interventions on autistic adults who have been subjected to them, as there have been reports of adverse effects.

The lack of an evidence base for older forms of Applied Behavior Analysis (ABA) has been mentioned by the Lancet Commission. However, there are other concerns that need to be addressed. In the past, unethical ABA practices included physical abuse and using the method in gay conversion therapy. Those who applied such practices included key developers of the methodology, such as Dr O. Ivaar Lovaas.^86,87^ This history needs to be openly admitted and the practices clearly renounced. There are other continuing ethical concerns,^88^ as well as issues with the evidence base of behavioral approaches more generally.

For example, Cochrane Review and meta-analysis of early-intervention ABA (early intensive behavioral intervention) found the overall quality of evidence low or very low.^89^ Seventy percent of ABA research has been reported to involve conflicts of interest, with less than 6% of the researchers declaring the conflicts.^90^ A recent US Department of Defence report on their Autism Care Demonstration program, which involves 47,000 certified ABA professionals and provides services to nearly 16,000 autistic people, mostly children and adolescents, expressed serious concerns about the lack of results from their ABA provision.^91^

Because of the emphasis on behavioral interventions, the Commission also appears to have ignored a number of more recently developed, promising possibilities.^15–19,44,92,93^ Researchers need to hear the many families that are seeking approaches that are more in alignment with the neurodiversity paradigm, and which are more oriented to the long-term well-being of autistic children than to their compliance with neurotypical behavioral norms.

In the light of the what has been stated earlier, elevating behavioral approaches above other therapeutic, habilitative, and educational methodologies to the status of medical treatments, and promoting them as treatments in LMICs, is considered by many autistic people a mistake of massive proportions.

## Call to Action

To illustrate our ongoing work, we wish to name a few examples that clinical researchers might want to be aware of, and that could function as starting points for further discourse.

In 2011, the Academic Autism Spectrum Partnership in Research and Education (AASPIRE) described a model for using Community Based Participatory Research (CBPR) to partner with autistic people, based on lessons from the first 5 years of their research collaboration.^27^ In 2017, a *Starter Pack for Participatory Autism Research*^94^ was published by the participatory Research Collective in the United Kingdom, followed in 2019 by a German *Checklist for Autism Friendly Research*.^31^

Also in 2019, the National Autistic Taskforce published *An Independent Guide to Quality Care for Autistic People*, describing quality provision for those with high support needs^95^; a Dutch autistic-led project published a report on *Onderzoeksagenda Autisme*, the most detailed study available on the research priorities of autistic people and other stakeholders^8^; and AASPIRE published “The AASPIRE practice-based guidelines for the inclusion of autistic adults in Research as Co-Researchers and Study Participants.” In 2021, The European Council of Autistic People presented preliminary results of a survey mapping the research priorities of autistic people in 12 languages.^96^ This year (2022), the Autistic Self Advocacy Network released a report titled “For whose benefit? Evidence, ethics, and effectiveness of autism interventions,” creating a template for a core set of underlying ethical principles for autism-related services.^97^

Although many ethical issues and questions remain unaddressed,^98^ it is encouraging to see an attempt at considering such ethical issues in clinical autism research and a call for “constructive collaborations” with autistic people and the wider autism community.^99^ However, although this is a welcome development, at times such collaborations have appeared merely performative.

We invite researchers and clinicians to join the critical conversation about ethics in autism research and services, and to actively include the voices of diverse autistic individuals and communities in their work. We wish to draw attention to power imbalances and lack of accessibility in such discourse. We lack platforms and channels to reach the research and clinical communities effectively. Current common practice that exacerbates the power imbalance is inviting individual autistic people to take participatory roles in research projects, representing their own “lived experience” only; in these roles, they are isolated, engaging with powerful organizations as individuals, often with very limited personal resources.

We call for the creation of shared platforms for continuing discourse on autism research at the global level, engaging autistic individuals as well as the organizations we have formed to advocate for our rights collectively.

## Ways Forward

We came together as an *ad hoc* committee to respond to the Lancet Commission. Although time pressures prevented us from extensive surveying of autistic stakeholders, our collective history of engagement in autistic communities and discourses allows us to state with some confidence that we represent the views and interests of a significant proportion of autistic people. We are aware of the limitations of the process we have engaged in, but we suggest that learnings from it could be used and expanded upon.

We hope to move toward a more permanent and systematically developed global body of autistic people, reaching larger numbers of autistic communities to establish a global pool of NGO representatives, community leaders, researchers, and scholars interested in engaging in autism research and related public discourse. This would allow widespread implementation of previous recommendations of involving community leaders as well as individuals representing the wider autistic community, equalizing some of the power imbalance.^27,28^ It would introduce the possibility of involving autistic people who are not interchangeable even when they disagree or criticize, being mandated and supported by their autistic and academic communities.

To break existing imbalances, it is also imperative to differentiate key stakeholders. In the case of autism research, stakeholders such as parents, caregivers, and clinicians are driving the research agenda. For decades, researchers have suggested therapies or interventions, often dismissing the views of those who have received them. Acknowledging autistic people as the key stakeholders is an essential and fundamental step forward. It could allow experience to be absorbed and transformed into knowledge to redefine the research strategy regarding autism. The research strategy itself should be community-oriented instead of disorder-oriented. An inclusive research strategy is a crucial component for the long-term positive results benefiting all stakeholders.

More autistic researchers are needed worldwide to bring more global attention to their viewpoints on autism. Autistic people worldwide should be encouraged to enter universities and undergraduate schools to discover their strengths in research. However, some universities can be reluctant to accept autistic students, or they may not have the expertise or resources to support them. Therefore, we need international grant programs to support higher education of autistic people.

Many methods and principles that can allow us to move forward already exist in the literature on participatory research. They have been successfully used in several countries, allowing us to recognize universal principles. For example, the “Toujishakenkyu” (Participatory study) method used by Japanese researchers could serve as one starting point for researchers everywhere to learn about autistic people's issues and insights.^100^

To give another example, collaborative consultation, if implemented with care, could be a positive tool. It has been suggested that participatory research, nevertheless, is still the exception rather than the standard, and that much of the participatory research that does take place is merely tokenistic.^101^

Thus, the most crucial question concerning participatory research at this point is not how to gain more knowledge about it, but how to consistently apply what we already know.

To increase accountability in autism research in general, it is necessary to create clear regulations on ethical engagement with the wider autistic community, using existing guidelines as the basis and aiming for global consensus. Increasing transparency in autism research, tackling conflicts of interest, and increasing autistic inclusion is crucial in developing accountability and trust. Autistic researchers, advocates, and community partners must be included at all levels of autism research, particularly in leadership.

Autistic people must be involved in setting the research agenda and have decision-making power in autism research, and not be merely tokenized. This inclusion is necessary to ensure that the research is aligned with the needs of autistic people. We must continue to establish and support groups and institutions that are aligned with the neurodiversity paradigm and have sufficient autistic representation. These groups must be consistently included in the discussion and decision making in autism research.

The power imbalance that currently allows interconnected non-autistic groups and individual researchers to control which research gets funded, how that research is conducted, and how research findings are reported must be challenged and rebuilt to a more equitable, inclusive system that centers on autistic people's needs and well-being.
